# Dual regulation of microglia and neurons by Astragaloside IV‐mediated mTORC1 suppression promotes functional recovery after acute spinal cord injury

**DOI:** 10.1111/jcmm.14776

**Published:** 2019-11-01

**Authors:** Jialiang Lin, Xiangxiang Pan, Chongan Huang, Mingbao Gu, Ximiao Chen, Xuanqi Zheng, Zhenxuan Shao, Sunli Hu, Ben Wang, Hao Lin, Yaosen Wu, Naifeng Tian, Yan Wu, Weiyang Gao, Yifei Zhou, Xiaolei Zhang, Xiangyang Wang

**Affiliations:** ^1^ Department of Orthopaedics The Second Affiliated Hospital and Yuying Children’s Hospital of Wenzhou Medical University Wenzhou Zhejiang Province China; ^2^ Key Laboratory of Orthopaedics of Zhejiang Province Wenzhou Zhejiang Province China; ^3^ The Second School of Medicine Wenzhou Medical University Wenzhou Zhejiang Province China; ^4^ Department of Orthopaedics Affiliated Hospital of Guilin Medical College Guilin Guangxi Province China; ^5^ Department of Orthopaedics The Second Affiliated Hospital of Zhejiang University School of Medicine Hangzhou Zhejiang Province China; ^6^ Chinese Orthopaedic Regenerative Medicine Society Hangzhou Zhejiang Province China

**Keywords:** Astragaloside IV, autophagy, microglia polarization, mTORC1, spinal cord injury

## Abstract

Inflammation and neuronal apoptosis contribute to the progression of secondary injury after spinal cord injury (SCI) and are targets for SCI therapy; autophagy is reported to suppress apoptosis in neuronal cells and M2 polarization may attenuate inflammatory response in microglia, while both are negatively regulated by mTORC1 signalling. We hypothesize that mTORC1 suppression may have dual effects on inflammation and neuronal apoptosis and may be a feasible approach for SCI therapy. In this study, we evaluate a novel inhibitor of mTORC1 signalling, Astragaloside IV (AS‐IV), in vitro and in vivo. Our results showed that AS‐IV may suppress mTORC1 signalling both in neuronal cells and microglial cells in vitro and in vivo. AS‐IV treatment may stimulate autophagy in neuronal cells and protect them against apoptosis through autophagy regulation; it may also promote M2 polarization in microglial cells and attenuate neuroinflammation. In vivo, rats were intraperitoneally injected with AS‐IV (10 mg/kg/d) after SCI, behavioural and histological evaluations showed that AS‐IV may promote functional recovery in rats after SCI. We propose that mTORC1 suppression may attenuate both microglial inflammatory response and neuronal apoptosis and promote functional recovery after SCI, while AS‐IV may become a novel therapeutic medicine for SCI.

## 1 INTRODUCTION

Spinal cord injury (SCI) is one of the most disabling central nervous system disorders that can lead to varying degrees of sensory and motor function loss. It has a major impact on the quality of life for patients.^1^ However, there is currently no recognized effective therapy to treat SCI. The pathological process of SCI is mainly composed of primary injury and secondary injury. Primary injury is mainly caused by the direct damage to the spinal cord, while it may trigger a series of pathology including inflammation, oxidative stress, ischaemia and local oedema, to induce secondary injury.^2^, ^3^, ^4^ Studies have shown that the secondary injury phase is an important time window for SCI therapy.^5^

Apoptosis of neurons, especially ventral horn motor neurons (VMN), may lead to the loss of motor function^6^, ^7^ and contribute to the secondary injury after SCI.^8^, ^9^ Besides, microglia‐mediated neuroinflammation also plays a crucial role in the expansion of secondary injury after SCI, and the degree of inflammation has a great impact on the recovery of motor function after SCI.^10^ Therefore, we propose that therapeutic strategies for SCI should focus on inhibiting neuronal apoptosis and inflammatory responses.

Autophagy is a conserved cellular degradation process that plays a critical role in cellular survival and homoeostasis. Recent studies revealed that autophagy may protect against apoptosis in neurons^11^; our group also showed that autophagy activators, such as rapamycin and metformin, may promote functional recovery in rats through apoptosis suppression.^12^, ^13^ Meanwhile, microglia polarization is an important target for regulating inflammation; studies have shown that microglia M2 polarization may attenuate inflammation after SCI.^14^ Intriguingly, both neuronal autophagy and microglial M2 polarization are regulated by mTORC1 signalling pathway.^15^, ^16^ Suppression of mTORC1 signalling pathway may not only promote autophagy in neurons but also induce M2 polarization in microglia; therefore, we hypothesize that mTORC1 suppression may have dual effects on inflammation and neuronal apoptosis and may be a feasible approach for SCI therapy.

Astragaloside IV (AS‐IV) is an extract of traditional Chinese medicine Radix Astragali. It has been shown to possess neuroprotective pharmacological activity by suppressing inflammation and oxidative stress in central nervous system diseases.^17^ More importantly, a previous study showed that AS‐IV could potently inhibit mTORC1 activity with less adverse effects.^18^ However, it is still not known whether AS‐IV could promote functional recovery after SCI. We propose that AS‐IV could reduce the inflammatory response and neuronal apoptosis so as to promote functional recovery after SCI by mTORC1 inhibition.

In this study, we found that AS‐IV may suppress mTORC1 signalling both in neuronal cells and microglial cells in vitro and in vivo. AS‐IV treatment may stimulate autophagy in neuronal cells and protect them against apoptosis through autophagy regulation; it may also promote M2 polarization in microglial cells and attenuate neuroinflammation. In vivo study, rats were intraperitoneally injected with AS‐IV (10 mg/kg/d) daily after SCI, behavioural and histological evaluations showed that AS‐IV may promote functional recovery in rats after SCI. We propose that mTORC1 suppression may attenuate both microglial inflammatory response and neuronal apoptosis and promote functional recovery after SCI, while AS‐IV may become a novel therapeutic medicine for SCI.

## 2 MATERIALS AND METHODS

### 2.1 Reagents

Antibodies specific for LC3B, p62, mTOR, Phospho‐mTOR, p70S6K, Akt, Phospho‐Akt, Cleaved caspase 3 (C‐caspase3), Bax, Cox‐2 and TNF‐α were purchased from Cell Signalling Technologies. Antibodies against iNOS, Bcl‐2, Iba1 and Arg‐1 were purchased from Abcam. The antibody of p‐p70S6K was obtained from Santa Cruz Biotechnology, and the antibody of CD16/32 was purchased from BD Biosciences Pharmingen. AS‐IV was purchased from MedChemExpress. Lipopolysaccharide (LPS) and tert‐butyl hydroperoxide (TBHP) were purchased from Sigma‐Aldrich. 3‐methyladenine (3‐MA) was purchased from Selleckchem. 4', 6‐diamidino‐2‐phenylindole (DAPI) was obtained from Beyotime. The polymerase chain reaction (PCR) primers were synthesized by Sangon Biotech, and other reagents used in the quantitative real‐time polymerase chain reaction (qPCR) were ordered from Takara Biomedical Technology. Horseradish peroxidase‐labelled secondary antibodies were purchased from Abcam, and AlexaFluor 594 and AlexaFluor 488 AffiniPure secondary antibodies were purchased from Yeasen.

### 2.2 SCI model and AS‐IV treatment

A total of 48 adult female SD rats (220‐250 g) were purchased from the SLAC Laboratory Animal Company. All experimental procedures were performed in accordance with Chinese Guidelines of Animal Care and Welfare, and this study was approved by Wenzhou Medical University Animal Care and Use Committee. Rats were divided randomly into three groups (the Sham group, the SCI group and the SCI + AS‐IV group). Before surgery, rats were anaesthetized with 1% (w/v) sodium pentobarbital (40 mg/kg, i.p.). Next, we cut‐off the muscles and ligaments around the T8 spinous process and performed a laminectomy at T8 vertebral level to expose the spinal cord. SCI was induced by a vascular clip (15 g forces; Oscar) to clamp the spinal cord for 1 min. The same surgical procedure was performed on the Sham group, but without compression of the spinal cord. After surgery, the bladders of rats were emptied manually twice a day. Besides, AS‐IV was injected (10 mg/kg i.p.) in the SCI + AS‐IV group (the AS‐IV group) immediately and given daily 10 mg/kg doses until they were killed. Simultaneously, the other groups were injected with an equal dose of saline.

### 2.3 Locomotion recovery evaluation

In our research, locomotor function of rats was assessed using the Basso, Beattie and Bresnahan (BBB) locomotor recovery scale, inclined plane test and footprint test. The BBB scale is a 21‐item scale that scored results ranging from 0 to 21 points in the open‐field test. For the inclined plane test, it was performed to assess functional improvement at each time‐point, and the maximum angle at which a rat could retain its position on a testing apparatus in two positions without falling for 5 seconds was recorded. The BBB scores and inclined plane test were performed on days 1, 3, 7, 14 and 28 after SCI. The footprint test was also performed and the footprints collected were scanned and evaluated. Each rat was observed by four evaluators who were blinded to the treatment.

### 2.4 Histological staining

On day 7 and 28 after SCI, the rats were anaesthetized and perfused with 0.9% NaCl through the heart, followed by fixation with 4% paraformaldehyde (PFA). The T7‐T9 spinal cord segment containing the lesion site was collected and fixed in 4% PFA for 48 hours, then embedded in paraffin for paraffin sections. Transverse sections with the thick of 5μm were prepared for histological staining. In order to observe the histological changes of each group, HE staining and Nissl staining were performed to paraffin sections according to the instructions of the manufacturer. All images were acquired using light microscopy (Olympus, Tokyo, Japan).

### 2.5 Cell culture and treatment

The rat‐derived microglial cell line (HAPI cells, kept in our laboratory) and the neuronal cell line (PC12 cells, purchased from the Shanghai Institute of Cell Biology) were cultured in Dulbecco's modified Eagle's medium (DMEM) supplemented with 10% foetal bovine serum (FBS), 100 units/mL penicillin and 100 μg/mL streptomycin. The cells were cultured at 37°C constant temperature incubator with a humidified atmosphere containing 5% CO_2_. To promote polarization to the M1 phenotype, HAPI cells were treated with LPS (1 μg/mL) for 24 hrs. In PC12 cells, oxidative stress, a common pathological mechanism of apoptosis, was induced by TBHP (100 μmol/L) for 4hrs. 3‐MA (10 mmol/L) was used to block autophagy. The treatment of AS‐IV (1 or 10 μmol/L) was used to study the effects of AS‐IV.

### 2.6 Cell viability assay

Cell viability was evaluated by the cell counting kit‐8 (CCK‐8; Dojindo Co, Kumamoto, Japan). PC12 and HAPI cells were seeded in 96‐well plates (5000 cell/cm^2^) and incubated in DMEM with 10% FBS at 37°C for 24 hours, respectively. Then, cells were treated with different concentrations of AS‐IV (0, 0.01, 0.1, 1, 10 and 100 μmol/L) for 24 hours, after washing the cells twice with PBS, 100 μL of DMEM containing 10 μL of CCK‐8 solution was added to the cells of each well. The plates were then incubated for approximately 1 hour, and the absorbance per well was measured at 450 nm using a microplate reader (Thermo, Rockford). Finally, we analysed the viability of cells under the influence of AS‐IV by absorbance.

### 2.7 Quantitative Real‐Time PCR

Total RNA was extracted from cells using TRIzol reagent (Invitrogen) following the manufacturer's protocol. 1 μg of total RNA from each group was used for reverse transcription. After the total RNA was reverse transcribed, the SYBR Green reagent (Bio‐Rad) was used in qPCR for quantification. And, the cDNA was amplified in a 10 μL reaction volume using the CFX96^®^ Real‐Time PCR System (Bio‐Rad). The PCR cycle began with template denaturation and hot‐start Taq activation for 3 minutes at 95°C, followed by 40 cycles of 95°C for 15 seconds and 60°C for 45 seconds. The normalized expression of the target gene was calculated using the comparative CT method, and a fold change was obtained from the equation $2^{-\Delta \Delta C_{t}}$ of each gene. Primers of iNOS, IL‐6, Arg‐1, Ym‐1 and GAPDH are listed in Table 1.

**Table 1:** Primers used for quantitative real‐time PCR analysis

Genes	Forward	Reverse
iNOS	AGACACATACTTTACGCCACTA	TCAAAGACCTCTGGATCTTGAC
IL‐6	TGCACTGTCAGAAAACAATCTG	CCAGAGCAGATTTTCAATAGGC
Arg‐1	GGGAAGGTAATCATAAGCCAGA	CCCAGATGACTTTTATGCGATG
Ym‐1	GAAGCATTTGGAGATGTGACTG	TAGTAGCAGACCAGTTTGTACG
GAPDH	ACGGCAAGTTCAACGGCACAG	CGACATACTCAGCACCAGCATCAC

### 2.8 Western blot analysis

Total protein was extracted from spinal cord tissues and cells. Then, the extracts were quantified by BCA kit (Beyotime) and were equilibrated to the same quality level. 12% SDS‐polyacrylamide gel electrophoresis was used to dissolve protein samples for the next phase that transferring them to polyvinylidene fluoride membranes (Millipore, 0.2 μm). Subsequently, the membranes were blocked for 2 hours using 5% skim milk in TBST (25 mM Tris, 150 mM NaCl, 0.05% Tween‐20, pH 7.5) and next incubated in the primary antibody solutions overnight at 4°C. Then, membranes were washed three times for 5 min with TBST and incubated with the relative secondary antibodies for 2 hours at room temperature. Finally, the bands were detected by a Chemi DocXRS + Imaging System (Bio‐Rad), and quantitative analysis was performed using Image Lab 3.0 software (Bio‐Rad).

### 2.9 Immunofluorescence

For in vivo studies, spinal cord sections were incubated in 3% H_2_O_2_ for 15 minutes at room temperature following by 30‐minute incubation in 10% bovine serum albumin containing 0.3% Triton X‐100 in a 37°C oven. After blocking, sections were incubated with the following primary antibodies: rabbit anti‐cleaved caspase 3 (1:200), goat anti‐Iba1 (1:200), rabbit anti‐Arg‐1 (1:200), rabbit anti‐NeuN (1:200) or mouse anti‐p‐p70S6K (1:100) at 4°C overnight. Then, sections were washed and incubated with corresponding secondary antibodies for 1 hour in a 37°C oven. DAPI was used to stain the nuclei of cells. For in vitro studies, cells were washed with PBS three times for 5 minutes and fixed in 4% PFA for 15 minutes at room temperature, following treated with 0.3% Triton X‐100 for 5 minutes. Next, 30‐minute incubation in 10% bovine serum albumin at 37°C was performed. Cells were incubated with primary antibodies at appropriate dilutions: rabbit anti‐p62 (1:400), rat anti‐CD16/32 (1:100) and rabbit anti‐Arg‐1 (1:200) at 4°C overnight. For secondary antibody incubation, the slides of cells were incubated with appropriate secondary antibodies for 1 hour. Then, the slides of cells were washed with PBS, stained with DAPI for 1 minute and then sealed with a coverslip. Images were captured using a fluorescence microscope (Olympus).

### 2.10 Statistical analysis

All data are expressed as mean ± standard deviation. All statistical analyses were performed using GraphPad Prism 7.0. Statistical significance was determined by one‐way analysis of variance (ANOVA) followed by Tukey's post hoc analysis. Besides, generalized linear mixed models were used to analyse between‐group differences in the BBB scores and inclined plane test results. The results were considered to be statistically significant at *P < .05 or **P < .01.

## 3 RESULTS

### 3.1 AS‐IV suppresses mTORC1 signalling and attenuates neuronal apoptosis in vitro

**Figure 1:**
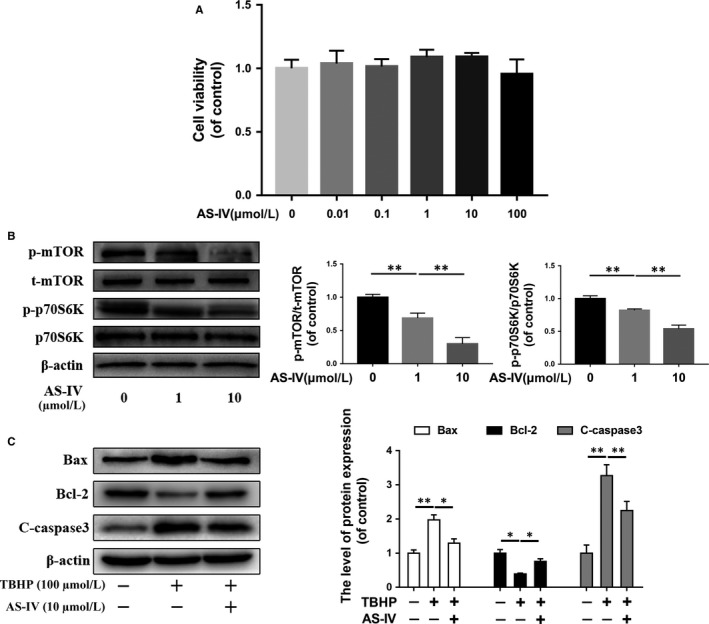
AS‐IV treatment suppresses mTORC1 signalling and prevents neuronal apoptosis in neuronal cells in vitro. (A) The Cytotoxicity of AS‐IV with different concentrations on PC12 cells was detected at by CCK8 assay after 24 h treatment. (B) Western blot results of mTORC1 signalling proteins expression in PC12 cells treated with different concentrations of AS‐IV on for 24 h. (C) Western blot results of apoptosis‐related proteins expression in PC12 cells, which were untreated, or treated with TBHP (100 μmol/L) alone, or treated with AS‐IV (10 μmol/L) and TBHP. Data are presented as the mean ± SD from three independent experiments. *P < .05 and **P < .01

PC12 cells were treated with different doses of AS‐IV (0, 0.01, 0.1, 1, 10 and 100 μmol/L) for 24 hours to assess the cytotoxicity of AS‐IV by CCK‐8. As shown in Figure 1A, the cell viability of PC12 cells was not significantly affected by AS‐IV at the dosage of ≤100 μmol/L after 24 hours of treatment. Then, we evaluated the effect of AS‐IV on mTORC1 signalling, we detected the phosphorylation levels of mTOR and p70S6K by Western blot, and we found the phosphorylation levels of mTOR and p70S6K reduced with increasing concentration of AS‐IV (Figure 1B). Next, we induced oxidative stress in PC12 cells using TBHP to assess the anti‐apoptotic effect of AS‐IV. Compared with the control group, the apoptosis‐related protein markers of Bax and cleaved caspase 3 were markedly increased while Bcl‐2 was reduced in TBHP group. However, the expression of Bax, cleaved caspase 3 and Bcl‐2 was restored in AS‐IV treated PC12 cells (Figure 1C). Moreover, TUNEL assay also confirmed that AS‐IV inhibited TBHP‐induced apoptosis in PC12 cells (Figure S1).

**Figure 2:**
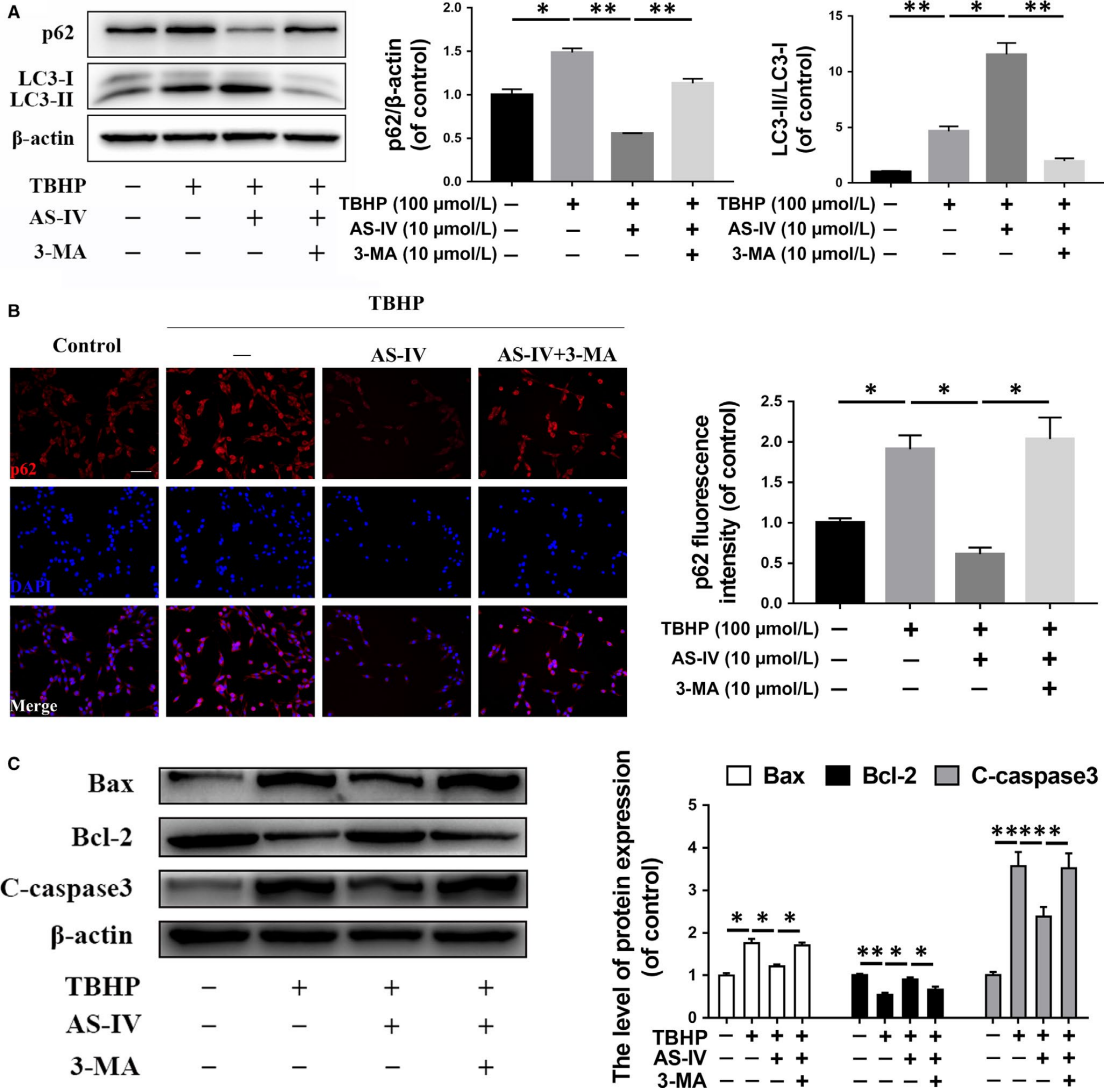
The anti‐apoptotic effect of AS‐IV in the neuronal cell is associated with up‐regulation of autophagic flux. PC12 cells were untreated, or treated with TBHP alone, or treated with AS‐IV and TBHP, or treated with TBHP and AS‐IV combined with 3‐MA (10 mmol/L). (A) The expression of p62 and LC3 was detected by Western blot in PC12 cells treated above. (B) Immunofluorescence staining and quantification analysis for p62 in each group as treated above. Scale bar = 50 μm. (C) The expression of Bax, Bcl‐2 and cleaved caspase 3 was detected by Western blot in PC12 cells treated above. Data are presented as the mean ± SD from three independent experiments. *P < .05 and **P < .01

To clarify the effect of AS‐IV on autophagy, we detected the expression of autophagy‐related protein, p62 and LC3, in PC12 cells. We found that the expression of p62 and LC3 was both increased in the TBHP‐treated group compared with the control group, while p62 expression level was decreased and LC3 was increased in the AS‐IV treated group compared with the TBHP‐treated group; the expression of p62 has also been confirmed by fluorescence staining (Figure 2B). These results suggested that although autophagy was activated, autophagic flux was inhibited by TBHP, while the autophagic flux was rescued by the treatment of AS‐IV (Figure 2A). Similarly, another Western blot assay showed that apoptosis‐related proteins were increased in the TBHP‐treated group compared with the control group, and they were decreased in the AS‐IV‐treated group (Figure 2C).

To further define the relationship between autophagy and neuronal apoptosis, we used 3‐MA, an autophagy inhibitor, to suppress autophagy. As predicted, autophagy activated by AS‐IV and anti‐apoptotic effects were both blocked by 3‐MA (Figure 2A,C). Together, these results suggest that AS‐IV treatment may suppress mTORC1 signalling and inhibit neuronal apoptosis through autophagy in vitro.

### 3.2 AS‐IV suppresses mTORC1 and promotes M2 polarization in microglia in vitro

**Figure 3:**
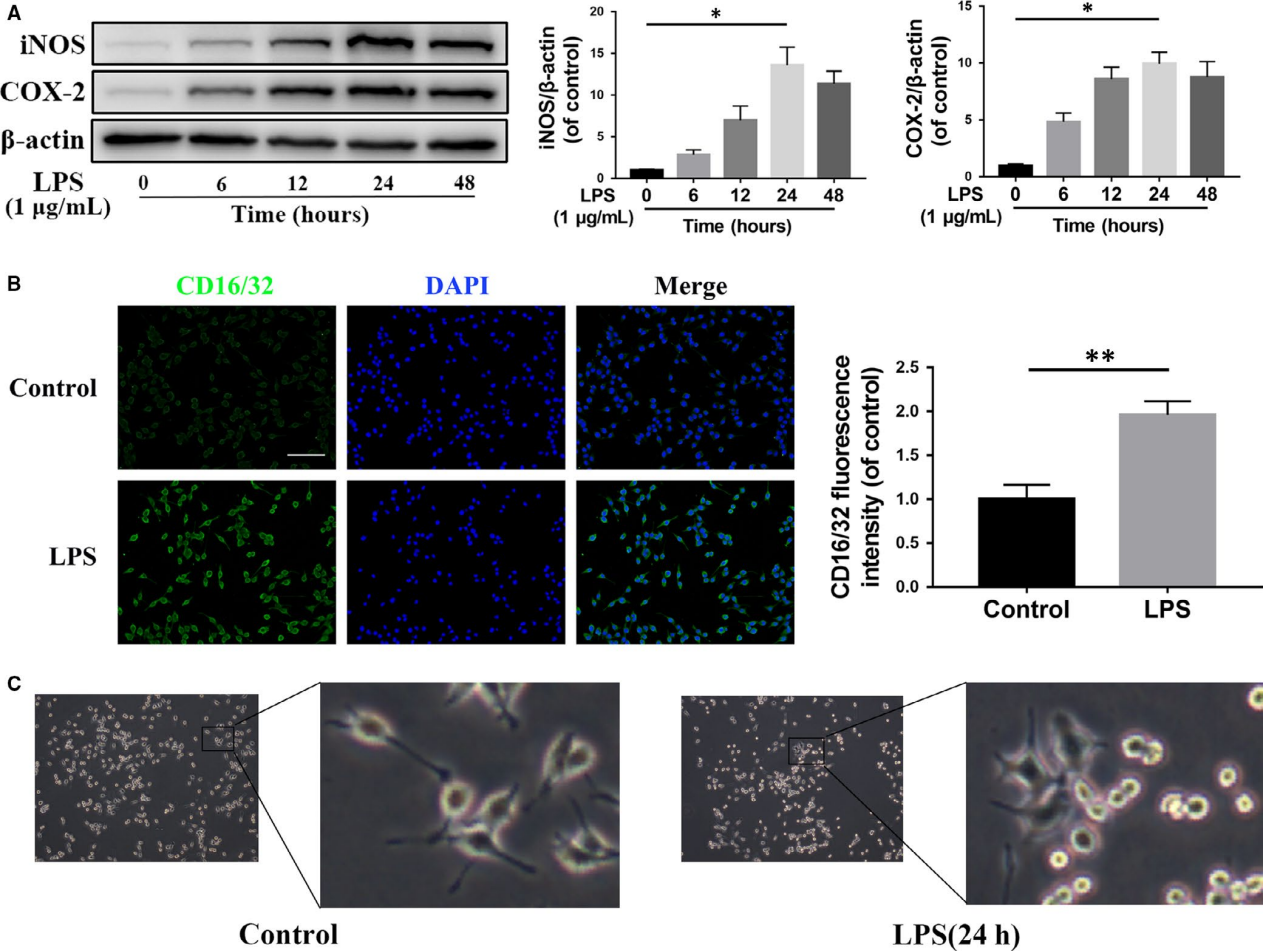
LPS induces M1 activation and increases pro‐inflammatory mediators release in microglia. (A) The expression of iNOS and COX‐2 detected by Western blot in HAPI cells treated with different time of LPS (1 μg/mL). (B) Immunofluorescence staining and quantification analysis for CD16/32 in HAPI cells treated with LPS (1 μg/mL) for 24 h. Scale bar = 100 μm. (C) Morphological change of HAPI cells with LPS (1 μg/mL) treatment for 24 h. Data are presented as the mean ± SD from three independent experiments. *P < .05 and **P < .01

We study the effect of AS‐IV on the polarization of microglia in HAPI cells in vitro. The Western blotting results showed that the release of pro‐inflammatory mediators, such as iNOS and COX‐2, were increased in a time‐dependent manner within 24 hours by the stimulus of LPS (Figure 3A), which indicated M1 type polarization of HAPI cells. Immunofluorescence staining of CD16/32, a marker of M1 microglia polarization, also showed that HAPI cells were significantly activated after 24 hours of LPS stimulation (Figure 3B). Moreover, as shown in Figure 3C, an amoeba‐like morphological change in HAPI cells also suggested activation of cells after LPS stimulation for 24 hours.

**Figure 4:**
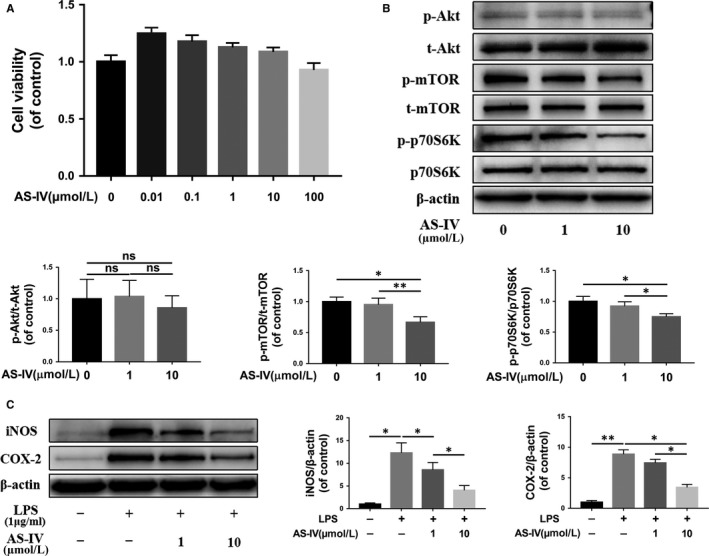
AS‐IV suppresses mTORC1 signalling pathway and pro‐inflammatory mediators release in microglia. (A) The cell viability of HAPI cells treated with AS‐IV at different concentrations for 24 h was evaluated by CCK‐8 assay. (B) The expression of mTOR signalling pathway proteins under the 24 h treatment of different concentrations AS‐IV on HAPI cells. (C) Western blot results of iNOS and COX‐2 in HAPI cells. HAPI cells were untreated, or treated with LPS (1 μg/mL) alone, or treated with AS‐IV (1, 10 μmol/L) and LPS. Data are presented as the mean ± SD from three independent experiments. *P < .05 and **P < .01

Next, we evaluated the effect of AS‐IV on the polarization of microglia. Firstly, we detected the cell viability of HAPI cells under different concentrations of AS‐IV, and the CCK‐8 assay showed that AS‐IV had no significant cytotoxicity against HAPI cells at a concentration of less than 100 μmol/L (Figure 4A). Western blot results showed that the phosphorylation levels of mTOR and its substrate, p70S6K, were decreased under AS‐IV treatment in a dose‐dependent manner; however, the ratio of p‐Akt/Akt was not changed in the AS‐IV‐treated group compared with the control group (Figure 4B).

**Figure 5:**
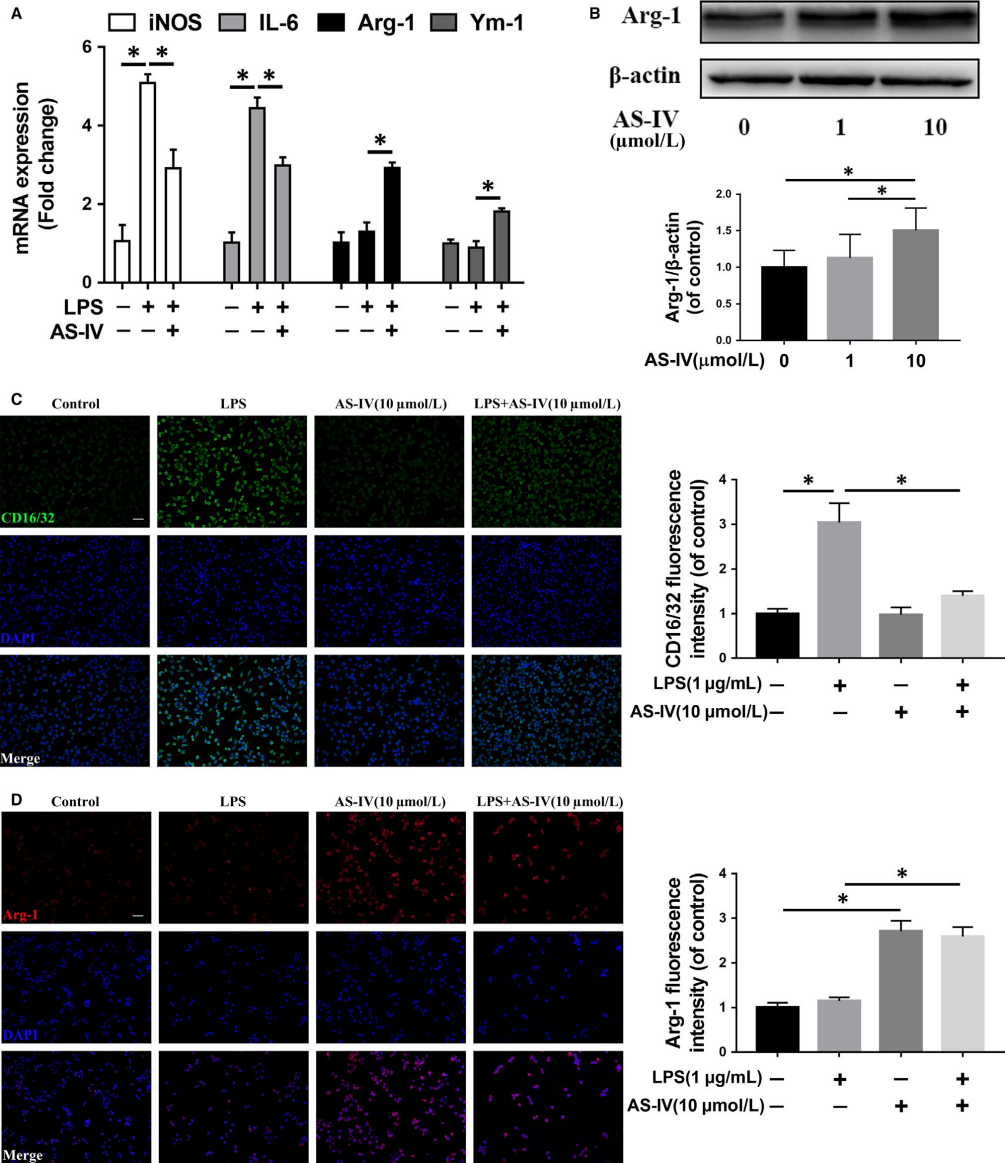
AS‐IV promotes M2 polarization in microglia. (A) The result of qRT‐PCR for iNOS, IL‐6, Arg‐1 and Ym‐1 gene expression levels in HAPI cells. (B) Western blot results of Arg‐1 in HAPI cells with the treatment of different concentrations AS‐IV for 24 h. (C,D) Immunofluorescence staining and quantification analysis for CD16/32 and Arg‐1 in HAPI cells. The cell was untreated, or treated with LPS (1 μg/mL) alone, or treated with AS‐IV (10 μmol/L) alone, or treated with AS‐IV (10 μmol/L) and LPS (1 μg/mL). Scale bar = 50 μm. Data are presented as the mean ± SD from three independent experiments. *P < .05 and **P < .01

To test the anti‐inflammatory effect of AS‐IV, we detected the expression levels of pro‐inflammatory factors, such as iNOS and COX‐2, and the results showed that they were distinctly inhibited by AS‐IV treatment in a dose‐dependent manner compared with the LPS‐stimulated group (Figure 4C). To further study the regulatory effect of AS‐IV on microglia polarization, we detected the mRNA expression of indicators of M1 and M2 polarization microglia under treatment of AS‐IV; it was found that AS‐IV inhibited microglia expression of pro‐inflammatory mediators induced by LPS and promoted the expression of Arg‐1 and Ym‐1, two markers for M2 microglia (Figure 5A). In Figure 5B, Western blot analysis indicated that proteins expression of Arg‐1 was increased after AS‐IV treatment in a dose‐dependent manner. Similarly, the immunofluorescence intensity analysis of HAPI cells showed AS‐IV down‐regulated CD16/32 expression and up‐regulated Arg‐1 expression compared with LPS‐stimulated group (Figure 5C,D). These results suggest that AS‐IV suppresses M1 polarization and promotes M2 polarization in microglia.

### 3.3 AS‐IV suppresses mTORC1 signalling in rats in vivo

**Figure 6:**
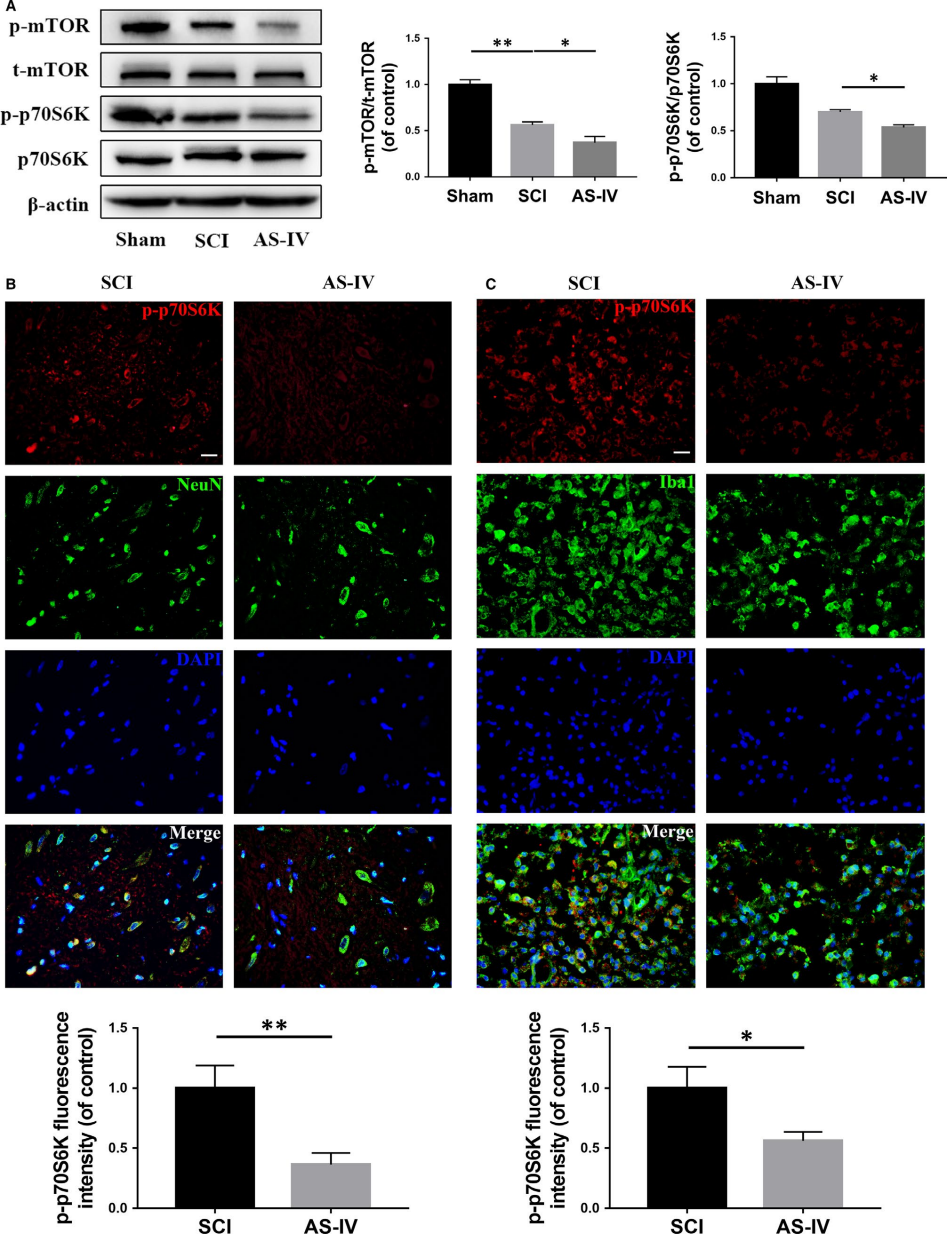
AS‐IV suppresses mTORC1 signalling in vivo in rats. A total of 3 groups of rats were given sham, SCI, SCI + AS‐IV treatment. (A) Western blot results of mTOR signalling proteins expression in each group of rats. (B,C) Double‐fluorescence staining and quantification analysis for p‐p70S6K (red)/NeuN (green) or p‐p70S6K (red)/Iba1 (green) of spinal cord tissue sections from SCI and SCI + AS‐IV treatment groups. Scale bar = 20 μm. Data are presented as the mean ± SD from three independent experiments. *P < .05 and **P < .01

To identify the effect of AS‐IV on mTORC1 signalling in vivo, we established SCI model in rats and examined the ratio of p‐mTOR/t‐mTOR and p‐p70S6K/p70S6K in spinal cord tissues by Western blot. We found both the phosphorylation levels of mTOR and p70S6K were reduced in the AS‐IV treated group compared with the SCI group (Figure 6A). After that, we performed immunofluorescence double staining on the cross‐section of spinal cord tissue slice; p‐p70S6K was co‐stained with NeuN or Iba1, which are markers of neuronal cells and microglia respectively, to reveal changes in mTORC1 signalling in neurons and microglia. As shown in Figure 6B, C, the fluorescence intensity of p‐p70S6K in the AS‐IV treatment group was significantly lower in NeuN and Iba1 positive cells compared with SCI group, suggesting mTORC1 signalling was inhibited both in neurons and microglia in vivo.

### 3.4 AS‐IV promotes locomotor function recovery after SCI in rats

**Figure 7:**
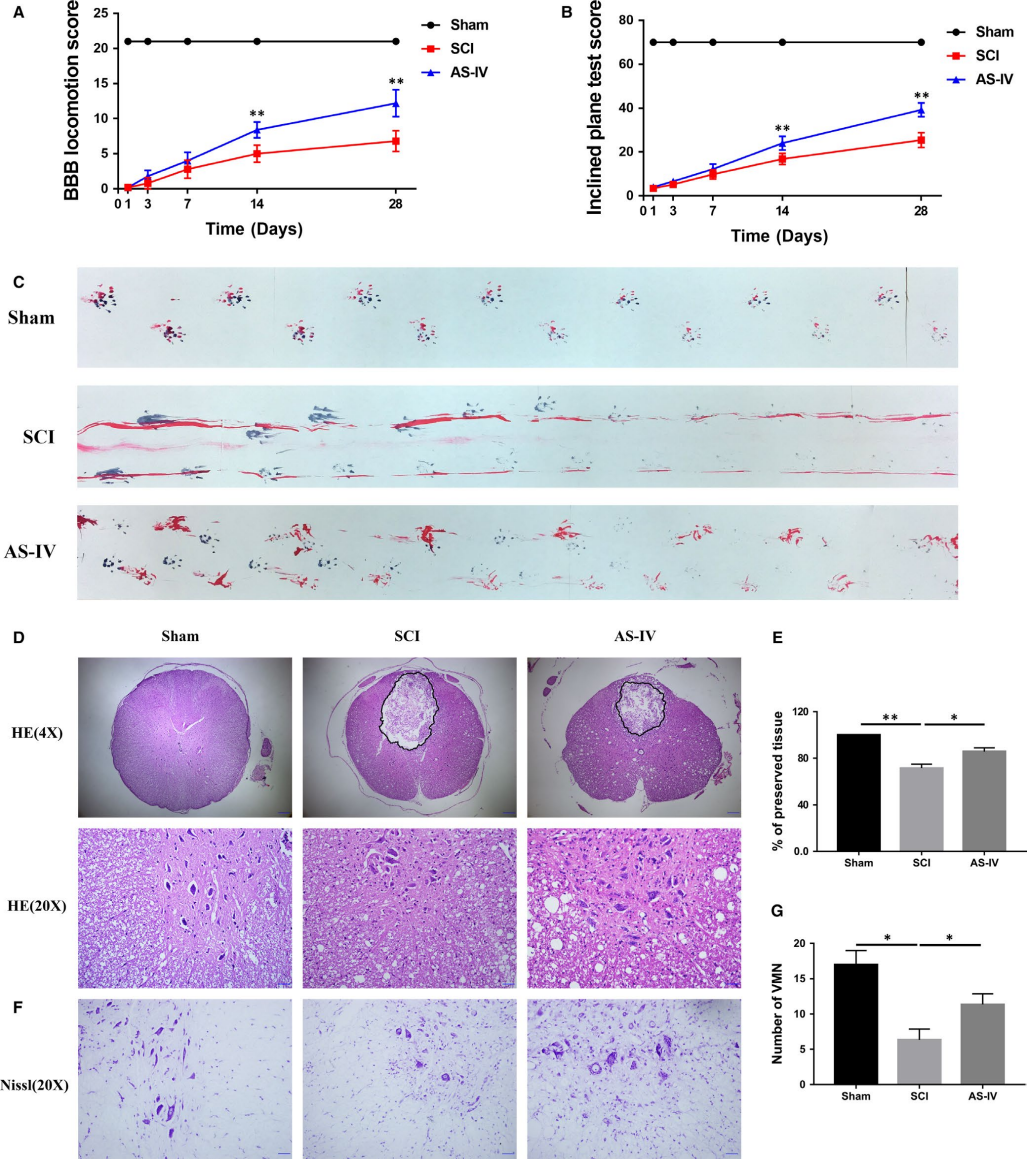
AS‐IV treatment promotes locomotor function recovery in rats after SCI. (A) The BBB locomotion scores and (B) inclined plane test scores of each group. (C) Footprint assay of different groups on the 14th day after injury. (D) HE and (F) Nissl staining of spinal cord tissue sections taken from the 28th day after SCI. Scale bars are 200 μm (4X) and 50 μm (20X). (E) Quantification analysis of the lesion cavity area in different groups. (G) Quantification analysis of the number of VMN. Data are presented as the mean ± SD from three independent experiments. *P < .05 and **P < .01

The above studies showed that AS‐IV possesses strongly neuroprotective and anti‐inflammatory effects in vitro; however, whether they can be beneficial for locomotor function recovery in vivo is still unknown. Therefore, we used three behavioural tests, the BBB locomotion score, the inclined plane test and footprint test, to evaluate motor function recovery in SCI rats. The results showed that all rats lost their hind legs mobility immediately after SCI compared with the sham operation group and gradually recovered in a time‐dependent manner. The results of the BBB score and inclined plane test showed that the AS‐IV treated group recovered better than the SCI group (Figure 7A,B). The footprint test vividly reflected differences in tracks of posterior limbs between different groups; rats in the SCI group showed distinct dragging of posterior limbs (red footprints) compared with the clear footprints of rats in the Sham group, whereas SCI rats with the treatment of AS‐IV displayed fairly consistent tracks of posterior limbs with little stumbling at 14 days after SCI (Figure 7C). Together, these results indicate that AS‐IV promotes functional recovery in SCI rats.

### 3.5 AS‐IV attenuates neuronal loss in SCI rats

**Figure 8:**
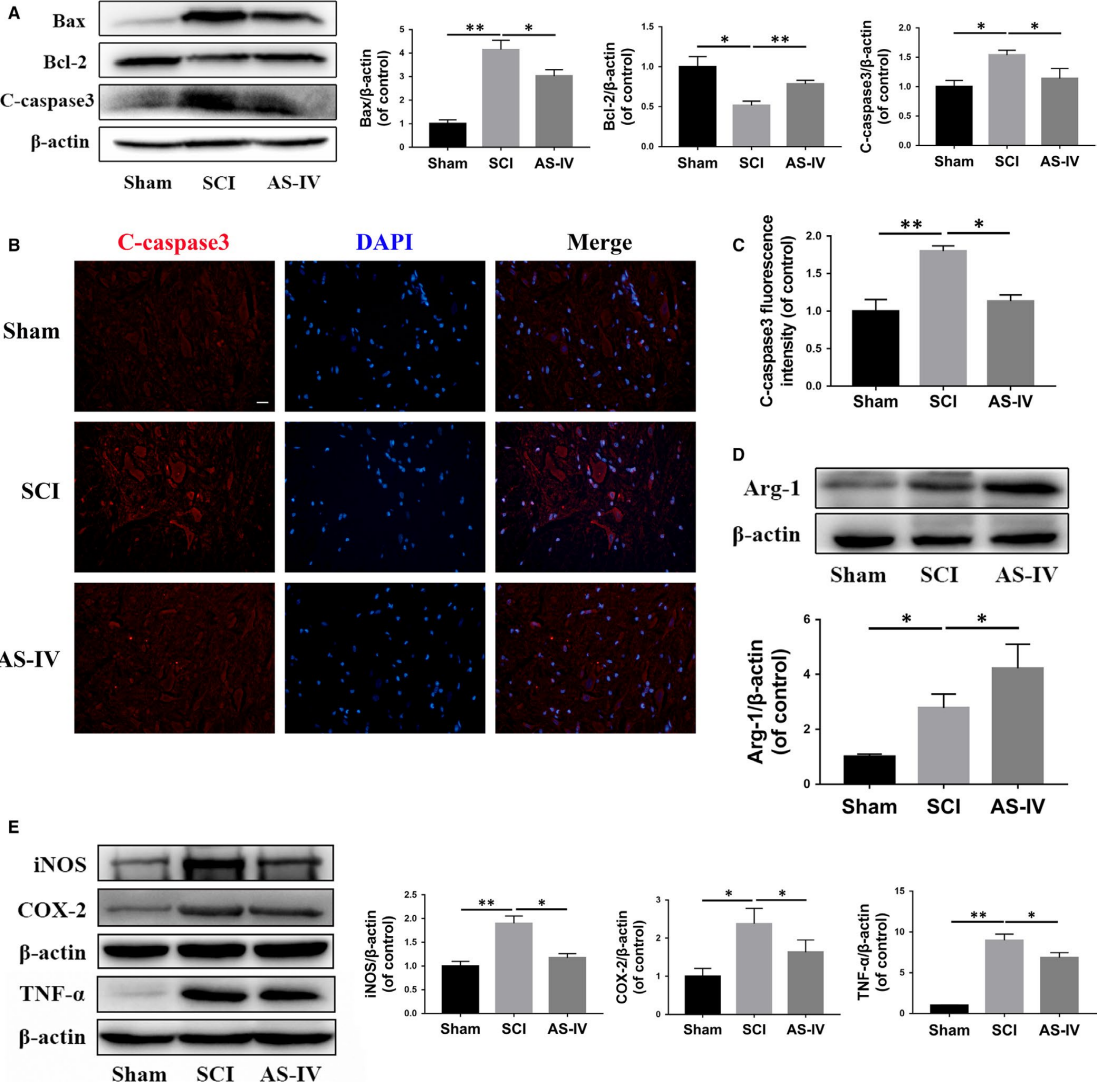
AS‐IV inhibits neuronal apoptosis and suppresses inflammatory response in SCI rats. (A) Western blot analysis of Bax, Bcl‐2 and cleaved caspase 3 in different treatment groups of rats. (B,C) Spinal tissue sections immunofluorescence staining and quantification analysis for cleaved caspase 3 in different treatment groups of rats. (D) The expression level and quantification analysis of Arg‐1 detected by Western blot in each group of rats. (E) Western blot results and quantification analysis of pro‐inflammatory mediators expression in each group of rats. Scale bar = 20 μm. Data are presented as the mean ± SD from three independent experiments. *P < .05 and **P < .01

To affirm the neuroprotective effect of AS‐IV in vivo, we observed neuronal retention in spinal cord tissue via HE staining and Nissl staining. As shown in Figure 7D,E, HE staining showed that central grey matter and dorsal white matter of SCI rats were seriously damaged compared with the Sham group, while AS‐IV treatment significantly attenuated the damage of spinal cord tissue. Also, we counted the numbers of ventral motor neurons (VMNs) via HE staining and Nissl staining, we found SCI induced massive loss of VMNs, while AS‐IV could attenuate its loss (Figure 7F,G). Next, we detected the expression of apoptosis‐related proteins at day 7 after SCI. The Western blot results showed that AS‐IV treatment effectively reversed the increase of caspase 3‐dependent apoptosis induced by SCI (Figure 8A). This was further supported by immunofluorescence staining of cleaved caspase 3; as shown in Figure 8B,C, the fluorescence intensity was decreased in the AS‐IV group compared with the non‐treated SCI group. Taken together, the results above suggested that AS‐IV could effectively attenuate caspase 3‐dependent neuronal apoptosis and restrain neuron loss in SCI rats in vivo.

### 3.6 AS‐IV promotes M2 polarization and suppresses pro‐inflammatory mediators release in SCI rats

To explore the effect of AS‐IV on microglia polarization in SCI in vivo, Western blot analysis was performed to detect the expression of Arg‐1. The results showed that AS‐IV promoted the expression of Arg‐1 compared with the SCI rats (Figure 8D). To further investigate the regulatory effect of AS‐IV on inflammatory response, we performed Western blot to observe the expression levels of pro‐inflammatory mediators, such as iNOS, COX‐2 and TNF‐α, at day 7 after SCI. The results showed that the pro‐inflammatory mediators above were distinctly increased in the SCI group compared with the Sham group; however, they were reduced in the AS‐IV‐treated group (Figure 8E). Collectively, these results indicate that AS‐IV promotes M2 polarization and inhibits the inflammatory response in SCI rats.

## 4 DISCUSSION

**Figure 9:**
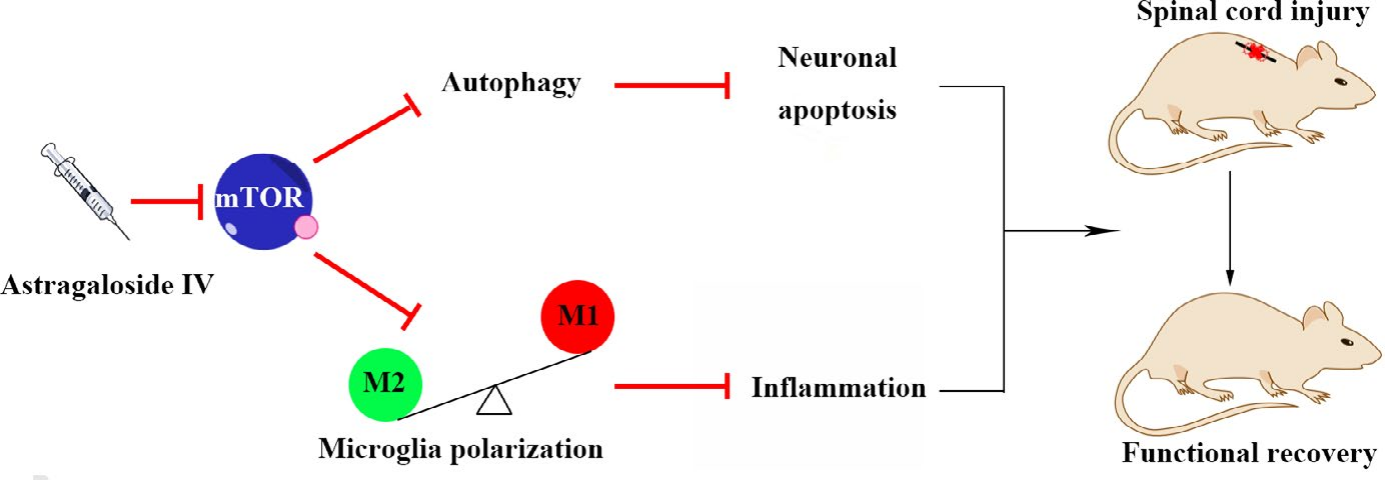
Schematic diagram of the effects of AS‐IV on SCI. AS‐IV treatment suppresses mTORC1 signalling pathway to regulate neuronal autophagy and microglia polarization, which promotes functional recovery of SCI rats

The incidence of SCI has soared particularly among young people, and it brings an enormous burden to the individual and society.^19^, ^20^ Thus, it is a priority to find an effective strategy to treat SCI. In recent years, a growing number of traditional Chinese herbal medicine ingredients have been tested in the treatment of SCI. AS‐IV is a Chinese herbal extract with low toxicity and minimal side effects. Many studies have demonstrated that AS‐IV possesses neuroprotective effects that can reverse or alleviate various central nervous system diseases, such as Parkinson's disease and cerebral ischaemia.^21^, ^22^ In the present study, we provide evidence that AS‐IV rendered a beneficial effect on neuroprotection against SCI. We found that (a) AS‐IV promotes autophagy and inhibits neuronal apoptosis in PC12 cells. (b) AS‐IV inhibits microglia M1 polarization and promotes M2 polarization in HAPI cells. (c) AS‐IV inhibits mTORC1 signalling in vitro and in vivo*.* (d) AS‐IV ameliorates secondary damage in the injured spinal cord and promotes locomotor function recovery after SCI in rats. Neuronal apoptosis and neuroinflammation are extremely important in the pathological process of secondary injury after SCI.^8^, ^23^, ^24^, ^25^ Numerous studies have targeted these two pathological processes for SCI therapy; however, barely any therapies could target both these two pathological processes. In our study, we found that AS‐IV possesses ‘one stone two birds’ potential, which not only inhibits neuronal apoptosis but also suppresses neuroinflammation through inhibiting the mTORC1 signalling pathway (Figure 9).

The mTORC1 signalling pathway plays a central role in regulating lots of essential cellular processes such as cell growth, proliferation and differentiation. Previous studies have shown that mTORC1 is a new therapeutic target for SCI.^26^, ^27^, ^28^, ^29^ In the process of apoptosis, mTORC1 suppression may promote autophagy, which may further suppress apoptosis, while mTORC1 suppression may also induce M2 polarization that may suppress neuroinflammation. In our study, we found that mTORC1 signalling was decreased in the SCI group when compared with the Sham group, while neuronal apoptosis and neuroinflammation were activated in the SCI group; however, when mTORC1 signalling was further suppressed by AS‐IV treatment (Figure 6 and Figure S2C), neuronal apoptosis and neuroinflammation were reduced, implying suppression of mTORC1 signalling is protective in SCI process and the internal suppression of mTORC1 signalling is not sufficient to combat impairment, which needs external assist. Both in vitro and in vivo results of our study (Figures 1B, 4B, 6 and S2) are consistent with previous reports and fully demonstrate that suppression of mTORC1 signalling has a beneficial effect on SCI therapy.

mTORC1 suppression could be achieved in multiple ways. Rheb1 is an isoform of Rheb that directly regulates the activity of mTORC1. Akt is another major upstream molecule to regulate mTORC1 signalling. It has been reported that AS‐IV may inhibit suppress mTORC1 signalling through Rheb.^18^ To answer whether AS‐IV may also inhibit mTORC1 signalling by regulating Akt, we assess Akt activity by detecting the phosphorylation of Akt. We found the phosphorylation level of Akt was not changed significantly with the treatment of AS‐IV (Figure 4B), implying that AS‐IV does not affect mTORC1 activity through the Akt axis. Some studies have also shown that AS‐IV can activate the AMPK to exert a series of biological effects,^30^, ^31^, ^32^ as AMPK is another upstream regulator to suppress mTORC1,^33^ and it is possible that AS‐IV may suppress mTORC1 signalling through AMPK other than Rheb.

Autophagy is an important response after mTORC1 signalling pathway suppression. Groups including ours have demonstrated that activation or restoration of autophagy may promote spinal cord repair after SCI.^12^, ^13^, ^34^, ^35^ The results of this study showed that although autophagy was activated in TBHP‐treated PC12 cells, autophagic flux was blocked (Figure 2A), suggesting a particular part of the autophagy process is impaired. The accumulation of p62 implied that the exception may occur in the degradation process, while lysosome is the key organelle responsible for the autophagic degradation process. Liu et al reported that lysosomal function was rapidly inhibited after SCI,^36^ which may explain the accumulation of p62 after SCI. The transcription factor EB (TFEB) is a master gene for lysosomal biogenesis,^37^ and it is regulated by mTORC1 signalling pathway.^38^ Therefore, we propose that AS‐IV mediated mTORC1 suppression may promote lysosomal biogenesis through TFEB, suggesting AS‐IV may have the potential of lysosome function regulation.

Microglial polarization plays a decisive role in the regulation of neuroinflammation after SCI. Generally, microglia can be classically activated into M1 (pro‐inflammatory) phenotype or alternatively activated into M2 (anti‐inflammatory) phenotype.^39^, ^40^ The polarization of both M1 and M2 affects the outcome of the inflammatory response, which may lead to either tissue damage or repair after SCI. Studies have found that a large number of microglia/macrophages polarized into M1 phenotype after SCI and secreted lots of pro‐inflammatory mediators, which were not conducive to the repair of SCI.^41^, ^42^, ^43^, ^44^ Therefore, we believe that proper anti‐inflammatory is necessary after SCI, and it is a good choice to regulate the microglial M2 polarization to exert anti‐inflammatory effects. In our study, we found that AS‐IV promoted microglia M2 polarization by inhibiting the mTOR signalling pathway, and it could effectively alleviate the inflammatory response after SCI (Figures 4, 5, 8C,D).

AS‐IV has been reported to have protective effects on cerebral ischaemia‐reperfusion injury as well as the integrity of the blood‐brain barrier.^45^, ^46^, ^47^, ^48^, ^49^, ^50^ With the low toxicity, AS‐IV may be expected to be applied to the clinic for the treatment of central nervous system injury in the future. Previous studies have also shown that rapamycin, a classical mTORC1 inhibitor, could exert neuroprotective effects in SCI.^28^, ^51^, ^52^, ^53^ Compared with these mTORC1 inhibitors, such as rapamycin, AS‐IV could effectively suppress mTORC1 signalling pathway with mild adverse effects.^18^ However, mTORC1 is still a key signalling in the regulation of cellular metabolism, and its suppression may bring a series of adverse reactions.^54^ In our study, AS‐IV was injected intraperitoneally, although therapeutic effects were shown in the study, the long‐term side effects of mTORC1 suppression by AS‐IV are still unknown. Nowadays, local delivery of drug with sustained release ability has become a new trend for SCI therapy.^55^, ^56^, ^57^, ^58^ Thus, further studies are needed to find a more suitable delivery system for AS‐IV.

## 5 CONCLUSION

AS‐IV mediated mTORC1 signalling suppression may inhibit neuronal apoptosis through autophagy and inhibit neuroinflammation through microglial M2 polarization, which leads to locomotor function recovery in SCI rats. Our study indicates that AS‐IV may become a potential drug for SCI treatment.

## CONFLICT OF INTEREST

The authors declare that they have no competing interests.

## AUTHORS’ CONTRIBUTION

XYW, XLZ and JLL contributed to conceptualization; CAH contributed to methodology; XQZ contributed to software; MBG and XMC contributed to validation; XXP contributed to formal analysis; ZXS contributed to investigation; XXP and BW contributed to resources; SLH contributed to data curation; JLL contributed to writing—original draft preparation; XLZ and YFZ contributed to writing—review and editing; HL contributed to visualization; YSW, YW, WYG and NFT contributed to supervision; XYW and XLZ contributed to project administration; XYW contributed to funding acquisition.

## Data Availability

All original data used in this work will be made available upon request.
