# Supplementary figures and images for: Dual regulation of microglia and neurons by Astragaloside IV‐mediated mTORC1 suppression promotes functional recovery after acute spinal cord injury
Source: J Cell Mol Med. 2019 Nov 1;24(1):671–85. doi: 10.1111/jcmm.14776 (PMC6933381; doi:10.1111/jcmm.14776)

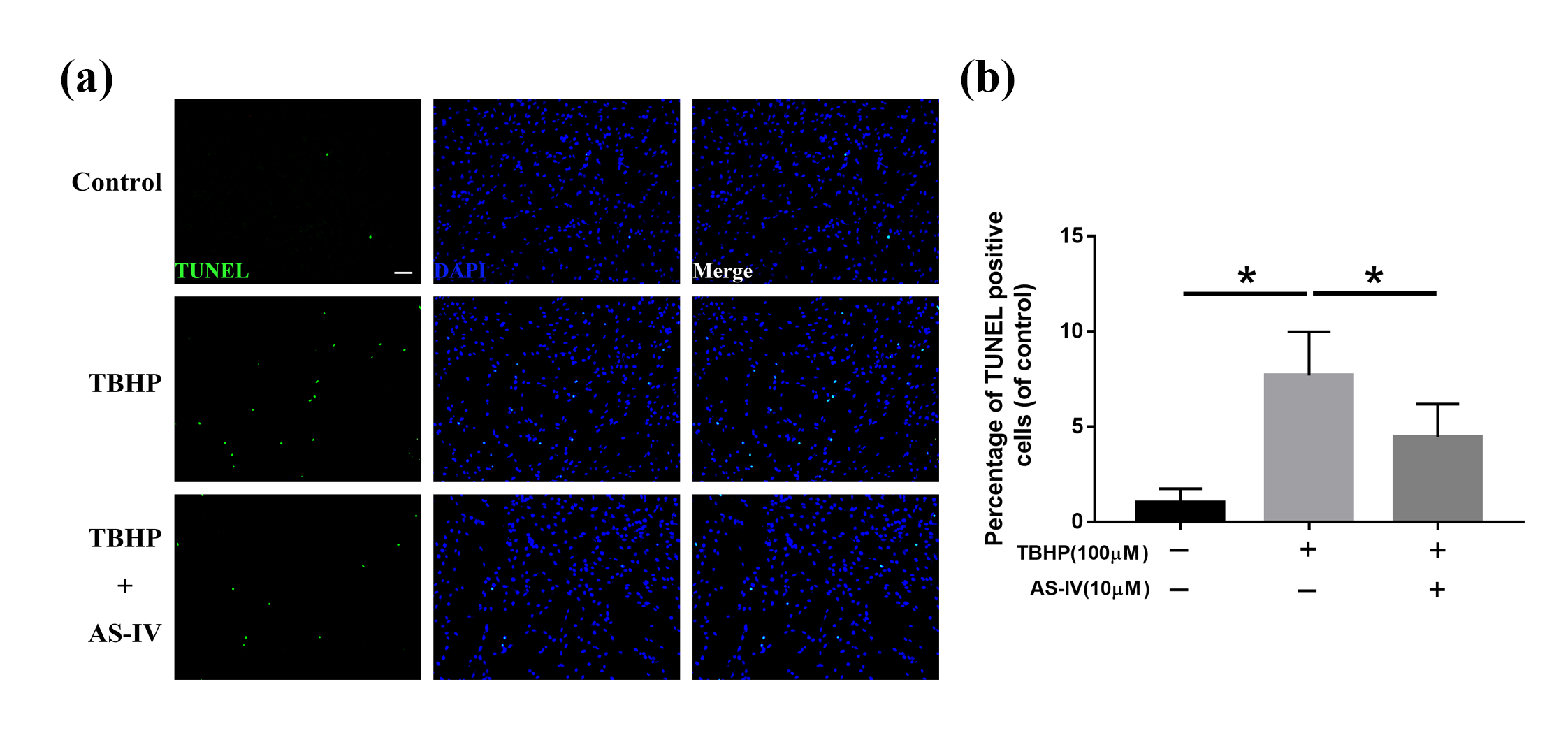

Supplement: Supplementary file 1 [file JCMM-24-671-s001.tif]

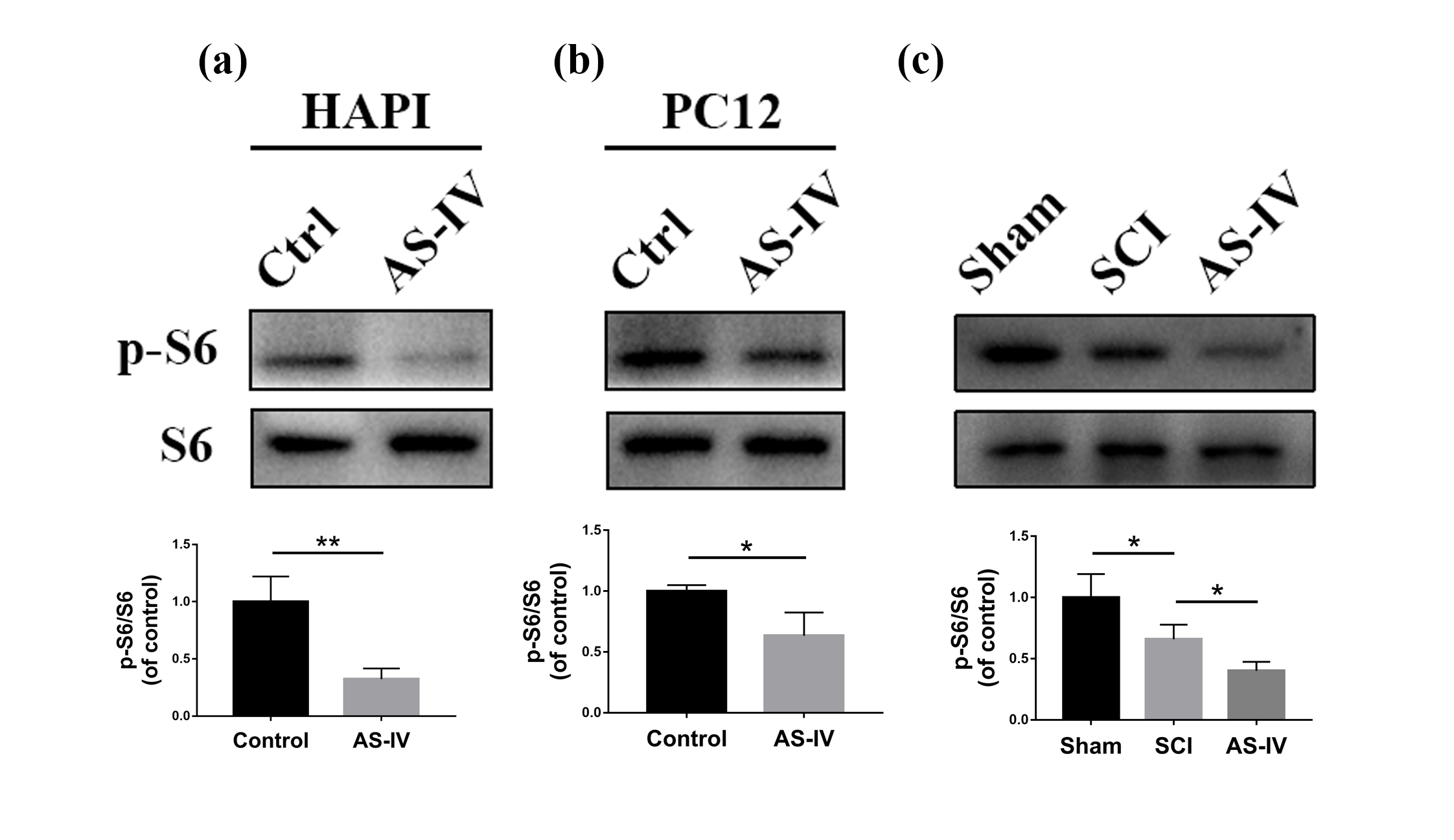

Supplement: Supplementary file 2 [file JCMM-24-671-s002.tif]
